# Single-cell transcriptome sequencing reveals heterogeneity of gastric cancer: progress and prospects

**DOI:** 10.3389/fonc.2023.1074268

**Published:** 2023-05-25

**Authors:** Gaohua Deng, Xu Zhang, Yonglan Chen, Sicheng Liang, Sha Liu, Zehui Yu, Muhan Lü

**Affiliations:** ^1^ Department of Gastroenterology, The Affiliated Hospital of Southwest Medical University, Luzhou, Sichuan, China; ^2^ Laboratory Animal Center, Southwest Medical University, Luzhou, Sichuan, China

**Keywords:** single-cell RNA sequencing, gastric cancer, tumor heterogeneity, oncogenesis, individualized therapy

## Abstract

Gastric cancer is one of the most serious malignant tumor and threatens the health of people worldwide. Its heterogeneity leaves many clinical problems unsolved. To treat it effectively, we need to explore its heterogeneity. Single-cell transcriptome sequencing, or single-cell RNA sequencing (scRNA-seq), reveals the complex biological composition and molecular characteristics of gastric cancer at the level of individual cells, which provides a new perspective for understanding the heterogeneity of gastric cancer. In this review, we first introduce the current procedure of scRNA-seq, and discuss the advantages and limitations of scRNA-seq. We then elaborate on the research carried out with scRNA-seq in gastric cancer in recent years, and describe how it reveals cell heterogeneity, the tumor microenvironment, oncogenesis and metastasis, as well as drug response in to gastric cancer, to facilitate early diagnosis, individualized therapy, and prognosis evaluation.

## 1 Introduction

Gastric cancer is a type of malignant tumor of epithelial origin and ranks fourth among all types of cancer worldwide (1). Due to its insidious early symptoms, most patients were diagnosed at an advanced stage, and the five-year survival rate is still less than 5% for patients with distant metastasis (2). Current treatment options, such as surgery, radiotherapy, chemotherapy, and targeted therapy, have achieved some clinical benefits, but still suffer from recurrence, metastasis, and drug resistance (3). Researchers are increasingly focusing on tumor heterogeneity in addressing these questions (4). In terms of histology, genomics, epigenetics, and other aspects, gastric cancer is increasingly recognized as one of the most heterogeneous tumor types (5). Identifying the intrinsic characteristics of gastric cancer tumors in different patients can help to pave the way for personalized therapy. However, several existing classifications, including Lauren’s (6), The Cancer Genome Atlas (TCGA) (7), and Asian Cancer Research Group (ACRG) (8), have contributed to the advancement of understanding gastric cancer, but due to numerous complex factors, it is still not enough to classify and treat all patients precisely. Single-cell transcriptome sequencing represents a new approach for studying the heterogeneity of gastric cancer.

Single-cell transcriptome sequencing, or single-cell RNA sequencing (scRNA-seq), has emerged as one of the technologies for next-generation sequencing (9). Rather than traditional bulk RNA sequencing (RNA-seq), which averages the gene expression levels of all cells, scRNA-seq transcripts of each cell, enable unprecedented recognition of the gene expression profiles of individual cells. In addition to discovering differences in cellular composition and characteristics, certain rare cell populations that are obscured by bulk RNA-seq are identified by using scRNA-seq (10). At the same time, by examining the tumor microenvironment at the level of individual cell, scRNA-seq has helped identify the valuable role of non-tumor cells in tumor development (11). Moreover, metastatic samples have been used to identify intrinsic features associated with metastatic for targeted therapy (12, 13). Analyzing pre- and post-treatment samples assists in discovering intrinsic mechanisms affecting drug response and paving the way for individualized therapy (14, 15). As scRNA-seq technology develops and costs go down, an increasing number of studies are being conducted on the technique. Currently, studies are being conducted on this technology in a number of tumor types, including gastric cancer, melanoma (16), lung cancer (17), liver cancer (18), and pancreatic cancer (19).

Here, we review and summarize the current basic procedure of scRNA-seq and analyze its advantages and limitations. We then present new insights into scRNA-seq in gastric cancer research, and discuss its challenges and application prospects based on the latest research results. Meanwhile, it is expected to provide some help in the diagnosis, treatment, and prognosis evaluation of gastric cancer.

## 2 Procedure of scRNA-seq

**Figure 1:**
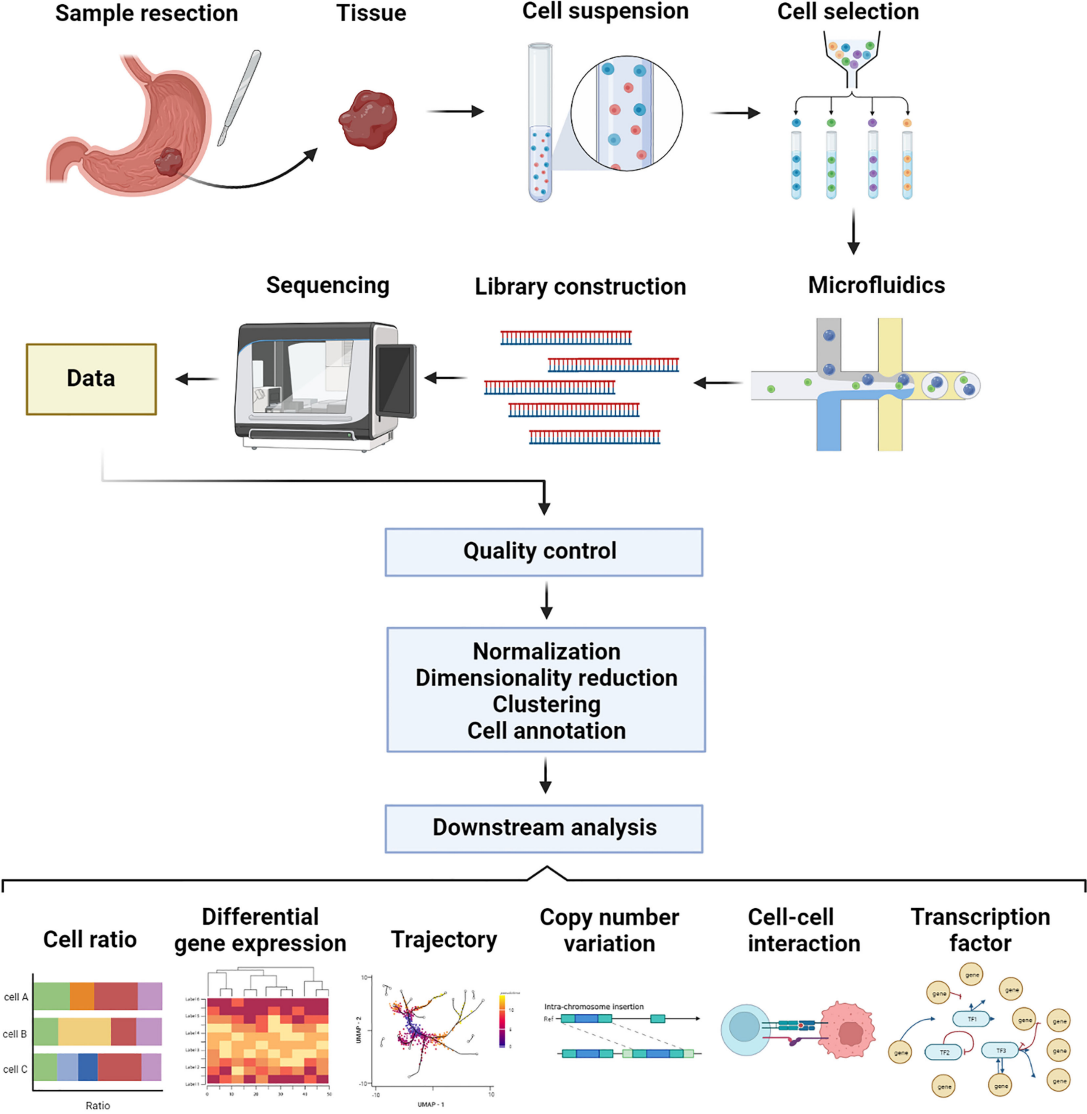
Procedure of single-cell RNA sequencing using microfluidics platform.

The scRNA-seq procedure consists of the following steps: single cell isolation, library construction, sequencing, and data analysis. **Figure 1** shows an overview procedure of scRNA-seq.

### 2.1 Single cell isolation

Samples of gastric cancer are typically obtained through endoscopic or surgical resection. After mechanical fragmentation of the tissue, enzymatic digestion was performed to obtain a single cell suspension (20). The enzymatic digestion time must be controlled. A short digestion time leads to incomplete digestion, resulting in cell clumps and doublet. Overly long digestion times can damage cells. To ensure that the analysis of sequencing results is not affected by altered cell state, it is important to evaluate the activity of single cell suspensions before sequencing, for example, by using trypan blue stain to determine cell viability. Moreover, specific cell types can be enriched and screened. Fluorescence-activated cell sorting (FACS) allows for the identification of fluorescently labeled cell-specific marker genes, which can be used for screening and enrichment of specific types of cells (20). Additionally, to prevent experimental failures caused by cell viability issues, sample processing and sequencing should be completed in a short period of time to minimize damage to cell viability.

### 2.2 Library construction and sequencing

RNA was obtained from cell lysis, followed by reverse transcription to obtain cDNA. The amplification and fragmentation of reverse transcription products are followed by ligating adapters to form a cDNA library (21). The library is constructed, and then is sequenced. Current sequencing methods can be divided into microwell-based methods, such as Smart-Seq2 (22), and droplet-based methods (23, 24), such as 10X Genomics (25). The following is a description of the most commonly used methods, Smart-Seq2 and 10X Genomics. Smart-Seq2 improves the capture of shorter transcripts and identifies transcriptional modifications, such as gene shearing and allelic expression, when full-length transcripts are sequenced (24), but the number of cells processed is limited. The 10X Genomics system uses microfluidics-based approaches to sequence genes by the 5’ or 3’ ends, which can process up to thousands of single cells per second, enabling faster sequences of samples with larger number of cells (26). It is more suitable to sequence samples with a larger number of cells. Unique molecular identifiers (UMIs), which are short barcodes attached to transcripts by 10X Genomics before amplification, allow 10X Genomics to avoid counting the same reverse transcription products twice. The expression level can be more accurately evaluated by reducing quantitative bias during amplification with UMIs in the 10X Genomics method (24, 27).

### 2.3 Data analysis

In data analysis, raw expression matrixes are processed, as well as downstream analysis. The processing of the original expression matrix, includes cell filtering, normalization, dimensionality reduction, clustering, and data integration (28). Data must be preprocessed. The use of cell filtering can reduce the interference caused by low-quality cells. Examples include poorly active cells that express high levels of mitochondrial genes and the artifacts caused by doublet. Dimensional catastrophe can be alleviated through dimension reduction. Using clustering, cells with similar characteristics were grouped together for annotation and further analysis. As single-cell sequencing involves batch effects and technical noise, integration is essential to reduce them so that they can be distinguished from biological differences.

Annotation of cells is typically performed before downstream analysis. Cell annotation in data analysis is a challenging task. The large number of cells make it difficult to annotate cells one by one. The current cluster-based cell annotation method assumes that all cells within a cluster are of the same type. However, annotation of new cell types will be difficult due to the lack of a uniform standard. Fortunately, the Human Cell Atlas provides a powerful guide for determining cell types (29). Additionally, cell marker genes reported in the available literature and machine learning methods can also be used to identify cell types (30).

The downstream analysis consists of a variety of procedures, such as cell abundance, differentiation gene expression, trajectory analysis, copy number variation (CNV) analysis, cell-cell interaction, and transcription factor analysis (31). For the cell ratio, the composition varies among patients at the level of single cells, demonstrating heterogeneity between them. Analysis of the differentiation gene expression and functional enrichments across each cell subpopulation showed their cell states and functions (32). Furthermore, some analysis tools can be used to perform various types of analyses. With tools such as Monocle (33) and Slingshot (34), trajectory analysis can demonstrate the dynamic process of cellular states, such as plasma cells, which have different stages of maturation in gastric cancer (35). Biological mechanisms underlie the transformation process and can be traced to cellular characteristics. InferCNV (36) and copyKAT (37) analyses of copy number variation can provide insights into chromosomal variation and help to determine the malignancy of tumor cells (16). Based on the expression levels of ligands and receptors, CellChat (38) and cellphonedb (39), for example, can assess in the construction of intercellular communication, which is a great resource to understand the complex interactions between cells and the role they play. Taking an example, the stromal cells in diffuse gastric cancer interact more frequently with other cell types (40), which may play an important role. By screening transcription factors and rebuilding gene regulatory networks, SCENIC (41) can enhance our understanding of tumor molecular regulation mechanisms at the level of individual cells.

ScRNA-seq profiles the transcriptome at the level of single cell, uses a variety of analytical methods, and is exceptionally advantageous for identifying gastric cancer heterogeneity. The sequencing depth of scRNA-seq, however, limits it to detecting only genes with relatively high expression abundance. Increased sequencing depth will allow more genes to be detected, but the ensuing increase in data volume and technical noise will make the algorithm more challenging (42). Although improvements to single cell technology are still needed, the constant development of single-cell RNA sequencing is expected to resolve the aforementioned issues.

## 3 ScRNA-seq reveals heterogeneity of gastric cancer

Increasingly, scRNA-seq is being used in gastric cancer studies due to its advantages and its achievements in other tumor studies. Samples for scRNA-seq studies of gastric cancer are currently available from a variety of sexes, disease stages, pathological types, and molecular subtypes. **Table 1** shows a summary of single-cell sequencing data currently available for gastric cancer research.

**Table 1:** A summary of the studies on gastric cancer using single-cell RNA sequencing.

Sample Type	Data Accession Number	Species	Contribution	Reference
GC, Normal, PC	GSE183904	Human	A high level of expression of INHBA and FAP in subpopulations of cancer-associated fibroblasts is associated with increased staging	(35)
DGC, Normal	GSE167297	Human	It is associated with an enrichment of CCL2 transcripts in inflammatory endothelial cells and fibroblasts in diffuse gastric cancer between the superficial and deep layer samples	(40)
NAG, CAG, IM, EGC	GSE134520	Human	A single-cell transcriptome atlas for gastric premalignant and early-malignant lesions, which spanned the cascade from gastritis to early gastric cancer	(43)
GC, Normal, CG	HRA000051	Human	Molecular evidences for potential transition from gastric chief cells into MUC6+TFF2+spasmolytic polypeptide expressing metaplasia	(44)
GC, CAG, IM, Normal	GSE150290	Human	Gastric cell landscape of intestinal gastric cancer and diffuse gastric cancer	(45)
HAS	HRA000077	Human	Adenocarcinomatous component and hepatocellular-like component of the same HAS tumor originate monoclonally, and HAS is likely to initiate from pluripotent precursor cells	(46)
HDGC	PRJEB41577	Mouse	The differentiation trajectory of squamous cells was shifted in HDGCs with Cdh1 inactivation	(47)
GC cell lines	GSE142750	Human	Heterogeneity of single cell transcriptome characteristics of gastric cancer cell lines	(48)
Ascites and cerebrospinal fluid	GSE140182	Human	Macrophages in malignant ascites of gastric cancer have strong non inflammatory properties	(49)
GC, Normal, PBMC, Blood	GSE172131	Human	Tumor infiltrating Tregs exhibit activated and effector states	(50)
AGC	EGAS00001004443	Human	Patient of gastric adenocarcinoma with peritoneal carcinomatosis was classified into two subtypes, the gastric-dominant and GI-mixed. Survival time for the former is shorter	(51)
GC, Normal	GSE158631	Human	Discovered some GC lymph node metastasis marker genes as well as potential gastric cancer evolutionary driving genes	(52)
GC, Normal, Metastasis	GSE163558	Human	Several subclusters of malignant epithelial cells were observed with invasion features, intraperitoneal metastasis propensity, epithelial–mesenchymal transition induced tumor stem cell phenotypes, or dormancy-like characteristics	(53)
CTCs	DRA011720	Human	A majority of gastric CTCs showed epithelial-mesenchymal transition	(54)
GC, Normal	CNP0001041	Human	The cytotoxicity and proliferation of T cells were decreased, immune pathways were downregulated, and angiogenesis pathways were activated in tumor cells and endothelial cells	(55)
GC, Normal	PRJEB45598	Human	TME remodeling was associated with response to first-line fluoropyrimidine and platinum chemotherapy	(56)
GC, Normal	PRJEB40416	Human	Compared to non-responders, responders of MSI-high patients treated with pembrolizumab had higher levels of T cells and NK cells	(57)
GC	GSE152888 GSE156725	Mouse	Immunotherapy for tumors can be guided by the deep immunological phenotyping	(58)

### 3.1 Cell heterogeneity

In normal gastric mucosa, there are a variety of cells, including pit cells, enteroendocrine cells, parietal cells, neck cells, chief cells, goblet cells, stem cells, immune cells and stromal cells (59). More complexity will be found in gastric cancer tissues (60). ScRNA-seq identifies the transcriptome at the level of single cell and has unrivaled advantages for uncovering cell heterogeneity, which provides new insights into discovering new biomarkers, diversity of tumors, and lineage compositions. According to the Correa hypothesis, gastric cancer arises from chronic atrophic gastritis, intestinal metaplasia (IM), and ultimately gastric cancer (61). Based on the Correa hypothesis, a study of gastric lesions at different stages found that a subpopulation of secretory pro-genitor markers goblet cells expresses HES6 during intestinal metaplasia. A population of these cells may represent goblet cells at an early stage of differentiation, and HES6 will help identify precancerous lesions in high-risk patients. For early gastric cancer (EGC), the discovery of two new specific markers, SLC11A2 and KLK7, provides clues for the early detection of EGC (43). Gastric cancer cells can also be heterogeneous. The scRNA-seq discrimination of gastric cancer cells is expected to lead to the stratification of patients based on the characteristics of cancer cells. In the study by Zhang et al, all malignant cells were divided into five groups: C1 is low differentiation cells mainly from diffuse gastric cancer (DGC) samples, C2 is high differentiation cells mainly from intestinal gastric cancer (IGC) samples, C3 is a mixed type with medium differentiation cells between C1 and C2, C4 is an entity fundic gland-type GA (GA-FG-CCP), and C5 is Epstein-Barr virus-infected type. Subgroups vary in their degree of differentiation, which correlates with the prognosis of patients (44). Since sample sizes are limited, scRNA-seq-based stratification of gastric cancer remains to be confirmed in large-scale sequencing data and evaluated for clinical value in guiding treatment and prognosis of patients.

In addition, there is heterogeneity within subtypes of gastric cancer. Based on cell state and characteristics, scRNA-seq provides new insight into gastric cancer development mechanisms in transcriptional dynamics. Lauren classifications divide gastric cancers into intestinal, diffuse, and mixed types (62). A Lauren classification-based study compared the trajectories of epithelial cells in gastric cancers of the intestinal type and diffuse type, and showed the different carcinogenesis mechanisms. IGC shows dynamic changes from non-malignant to tumor cells, with IGC marker genes such as MUC13 and CDH17 expressing higher levels over time, and the risk of tumorigenesis increased accordingly, supporting the Correa hypothesis. In contrast, in DGC, the expression of markers genes of IM and IGC does not change with stage that according to cell lineage, and has no relationship with the pathological classification. The carcinogenic mechanism of DGC is different from IGC (45). In specific types of gastric cancer, transcriptional dynamics differed as well. In a study of hepatoid adenocarcinoma of the stomach (HAS), all epithelial cells from HAS expressed cancer-related genes, and showed a significant copy number of variations from common gastric cancer. Cancer cells of HAS may have been derived from pluripotent precursor cells, which then differentiated into corresponding epithelial cell lineages such as adenocarcinoma and hepatocyte-like components (46). Moreover, in animal models, scRNA-seq is also applicable. Hereditary diffuse gastric cancer (HDGC) is a cancer syndrome caused by inactivating germline mutations in CDH1, but its mechanism remains unclear. In a mouse organoid model of HDGC, deregulation of developmental transcriptional programs was found at the early stage of CDH1 deletion that are associated with differentiation of gastric squamous epithelial cells in mouse (47). All these studies demonstrate that scRNA-seq provides a powerful tool for research on the transcriptional dynamics and lineage compositions of gastric cancer.

Furthermore, new cell types were identified in large-scale scRNA-seq data. As an example, new cell types that express both endothelial cells and fibroblasts marker genes may be undergoing endothelial-mesenchymal transition (EMT) (35). A variety of gastric cancer cell lines also showed multiple clusters with differing characteristics (48). In part, this may explain why experimental reproducibility differs among cell lines.

In summary, gastric cancer shows cell heterogeneity. ScRNA-seq is a powerful tool for understanding the transcriptional dynamics and lineage compositions of gastric cancer.

### 3.2 Tumor microenvironment

All cells and their secretory products, along with the extracellular matrix, make up the tumor microenvironment (63). Immune cells and stromal cells contribute significantly to tumor invasion, metastasis, and drug resistance as non-tumor cells in the microenvironment (64). While bulk RNA-seq can be evaluated for immune infiltration with deconvolution, some limitations remain (65). In microenvironments, scRNA-seq can distinguish cell types at the level of single cell, and it has great advantages for studying the “dialog” and regulatory mechanisms between cells. As a major component of the tumor microenvironment, immune cells take part in tumor immunity response processes. In a study of ascites from patients with advanced gastric cancer (AGC), macrophages in GC malignant ascites have non-inflammatory characteristics compared with *in vitro* macrophage transcripts. Cancer cells interact with tumor-associated macrophages (TAMs) *via* IL1B-IL1R2 ligands and receptors, thereby inhibiting inflammatory signaling, and promoting tumor growth (49). Furthermore, tumors contain tertiary lymphatic structures (TLSs), which function as organized aggregates of immune cells, involved in the immune response process in tumors (66). A study found that tissues with mature tertiary lymphatic structures (mTLSs) contain more types of immune cells (67). This may result in more aggressive antitumor responses. However, previous studies have shown that regulatory T cells (Tregs) inhibit antitumor responses (68). ScRNA-seq also shows that immunosuppression is associated with increased Tregs in tumor microenvironment of gastric cancer (69). In another study, tumor microenvironment containing TNFR2-positive Tregs was associated with poor prognosis (50). An assessment of Tregs levels may provide prognostic information. There is a need to pay more attention to the interaction between tumor cells and immune cells, which will facilitate the development of new immunotherapy strategies. A study by Kumar et al. found that KLF2 expressed in epithelial cells was associated with the recruitment of plasma cells in IGC (35). KLF2 may be a potential therapeutic target for recruiting plasma cells in DGC based on their role in gastric cancer (70).

In the microenvironment, stromal cells produce cytokines and chemokines, which affect tumor growth and invasion by promoting extracellular matrix formation and angiogenesis (60). ScRNA-seq analysis also confirmed the significance of stromal cells in tumors. In DGC studies conducted at different sampling depths, there may be a relationship between cell types and the depth level of tumors. Deeper layers were enriched with endothelial, fibroblast, and myeloid cells. In comparison with superficial tumor layers, deeper layers have a more intense cell-cell communication (40). An analysis of the microenvironment of cascade changes in gastric cancer also confirmed the importance of these cells (71). It is notable that the deep enrichment of CCL2-expressing stromal cells in DGC correlates with its invasive ability (40). CCL2 may be a potential target for DGC interference. A study by Kim et al. differentiated tumor-associated fibroblasts (CAFs) into three types based on gene expression profiles: inflammatory (iCAFs), myofibroblastic (myCAFs), and intermediate (inCAFs). One of them, iCAFs, is closely related to GC invasion and promotes the stemness of tumor cells, which can identify patients at high-risk of GC (45). A comprehensive study of the diversity of gastric cancer fibroblasts would benefit from this research. Furthermore, a large-scale sequencing study revealed that INHBA is a positive regulator of FAP in the fibroblast population, and that high expression of these genes in CAFs is associated with poor prognosis (45). As seen in the microenvironment of tumors, stromal cells are also diverse, and appear to be potential therapeutic targets and prognostic indicators.

To conclude, scRNA-seq offers new options for understanding the heterogeneity of tumor microenvironment, which will provide more opportunities for targeted therapies of gastric cancer.

### 3.3 Oncogenesis and metastasis

In terms of the mechanism of gastric carcinogenesis, there are still some controversies. Research suggests that long-term chronic inflammation induces intestinal metaplasia as a precursor to gastric cancer (72). Several studies have shown that the abnormal differentiation of chief cells will transform into neck cells, and the consequent development of spasmolytic polypeptide-expressing metaplasia (SPEM), which is associated with dysplasia and cancer (73). Using scRNA-seq, further evidence is presented for the role of SPEM in gastric cancer development. In a study by Zhang et al., cells expressing MUC6, TFF2, CD44, SOX9, and major histocompatibility complex class II genes were classified as SPEM cells. The results confirm SPEM at the level of single cell, suggesting a differentiation pathway between the chief cells and the neck cells, which in turn gives rise to SPEM cells (44). However, despite this, SPEM cells are not clearly defined. With the addition of scRNA-seq recognition, the definition of SPEM has been expanded. A study by Bockerstett et al. examined SPEM in a mouse model showed that the definition of SPEM should include TFFF2+Muc6+GIFF- metaplastic cells without mature principal cell transcripts, which may result in the same cancer risks as standard SPEM. Considering the complexity of the pathological mechanisms, the authors also examined the levels of SPEM transcript in drug-induced acute gastritis and chronic inflammation gastritis mouse models. The results show that SPEM cells are transcriptome-conserved across the two gastritis models (74). Another subsequent study by the same group revealed Gastrokine-3 (Gkn3) expression in the gastric body of chronic atrophic gastritis patients but not in healthy people. Both mouse models and human tissues have demonstrated the specificity of Gkn3 for SPEM recognition (75). Therefore, in chronic atrophic gastritis, identifying SPEM cells should include GKN3-positive cells in the gastric body, so that we can identify SPEM more accurately. SPEM is a type of metaplasia which associated with a risk of cancer, and its reversal may reduce that risk. A previous study confirmed the necessity of IL13 in metaplasia of chief cells (76). Using scRNA-seq, researchers found that mouse with gastritis with loss of IL4/IL13 signaling did not upregulate SPEM transcript levels. Following this treatment, SPEM was development were significantly reduced and reversed in mice with autoimmune gastritis treated with an IL13 antibody (77). It is possible to prevent and/or reverse atrophic gastritis to metaplasia through the inhibition of IL13 and its receptors. To reverse the metaplastic state and prevent the occurrence of gastric cancer is of great significance.

Clonal evolution suggests that tumor progression is also a dynamic process, and each stage has its own characteristics (78). Whether a tumor is progressing, metastasizing, or spreading to different organs, it exhibits inherent characteristics. Using scRNA-seq to analyze gastric cancer at different stages can reveal the characteristics of changes related to its development and outcome, which are critical for discovering new blocking targets and assessing prognosis. According to the findings of a study of peritoneal carcinoma (PC) in gastric adenocarcinoma patients, PC malignant cells are highly heterogeneous among patients, with most clustering by patient. Depending on the lineage status of malignant PC cells, different oncogenic pathways are involved in patient survival. Those tumor cells with gastric characteristics had shorter survival times and were predominantly enriched in oncogenic pathways, while those with enterocyte characteristics had longer survival times and were predominantly enriched in immune pathways (51). Hence, PCs with a variety of cellular characteristics may be useful in determining survival prognosis. An analysis of gastric cancer lymph node metastasis revealed the presence of ERBB2, CLDN11 and CDK12, as markers of lymph node metastasis, which may contribute to lymph node metastasis in gastric cancer (52). Aside from the peritoneum and lymph nodes, the liver, lung, and ovary are common metastatic organs for advanced gastric cancer. According to Jiang et al., a study of metastatic foci from different organs of gastric cancer found that malignant cells have invasion features, organ metastasis tendency, tumor stem cell phenotypes, and dormancy-like characteristics (53). These characteristics of malignant cells may be associated with metastatic propensity and recurrence. Together, scRNA-seq demonstrates the heterogeneity of metastases from gastric cancer.

Cells found in the circulatory system of tumor patients are known as circulating tumor cells (CTCs), which are thought to be associated with distant metastases (79). In a scRNA-seq study of gastric cancer CTCs, mesenchymal genes such as ZEB2 and SERPINE1 were highly expressed, possibly suggesting that they underwent EMT (54). Heterogeneity in gastric cancer has been further explored in these studies. In addition, tumor stem cells have also been implicated as a cause of progression, metastasis, and recurrence in gastric cancer (80). The use of scRNA-seq has provided new insights into cancer stem cells in some studies, including bladder cancer and liver tumors (81, 82). Using scRNA-seq, more evidence will be provide for cancer metastasis in gastric cancer stem cells.

In summary, scRNA-seq offers more possibilities for studying gastric cancer development and metastasis.

### 3.4 Drug response

For patients with advanced gastric cancer, effective treatment is particularly crucial given the low survival rates. A main treatment option for advanced patients is chemotherapy, and patients respond to drugs differently due to tumor heterogeneity (83). It is possible to discover potential mechanisms that affect the drug response through scRNA-seq by detecting changes in cells before and after drug administration. This has important implications for making “cold tumors” hot tumors. A study on neoadjuvant chemotherapy found that chemotherapy could remodel the microenvironment. As compared with the pre-treatment samples, the post-treatment samples showed impaired immune cells, but increased endothelial and fibroblast cells. The T cells demonstrated lower cytotoxicity and proliferation characteristics than the pre-treatment tumors, along with downregulation of immune pathways, and the angiogenesis pathway is activated in tumor cells and endothelial cells (55). Research on advanced gastric cancer has also shown immune remodeling during chemotherapy. It was found that pro-inflammatory genes and MHC class I antigen-presenting genes decreased after chemotherapy, as well as the expression of M2-type macrophage-related genes, indicating that macrophages after chemotherapy transformed from M2 to M1 cells. LAG3 was expressed by T cells in non-responders patients, which may be associated with drug resistance (56). It was also shown that immune remodeling occurs during the early process of chemotherapy in gastric cancer. Despite the small sample size, this study provides a good foundation for predicting how individual patients will respond to chemotherapy. A greater understanding of drug resistance requires the study of larger-scale data and the advancement of molecular mechanism research.

An emerging cancer treatment strategy involves immunotherapy. Several immune checkpoint blockade treatments have been approved as third-line therapies for advanced gastric cancer due to their favorable trends in patients, including pembrolizumab, nivolumab, avelumab, durvalumab, and atezolizumab (3). In a large-scale clinical trial of PD-L1 blockade therapy in metastatic gastric cancer, EBV-positive patients showed a good clinical response to PD-L1 blockade therapy. A better response to drugs is also observed in patients with microsatellite instability-high (MSI-H) (84). Although PD-L1 blockade treatment has shown promising results in clinical trials, some patients still do not respond and develop drug resistance. The scRNA-seq approach offers a new opportunity to discover potential mechanisms of drug resistance. According to a study of patients with MSI-H treated with pembrolizumab, responders had higher levels of T cells and NK cells than non-responders. The number of T cells and NK cells decreased in non-responders after two cycles of treatment, whereas stromal cells increased. In further analysis, a further study of T cell subsets revealed a significant increase in exhausted CD8+ T cells (57). Thus, failure to mobilize the immune system is associated with the lack of response to pembrolizumab in non-responders with MSI-H gastric cancer. The discovery of how to activate the immune response of non-responders will have revolutionary significance for tumor treatment.

Clinical problems associated with drug resistance can be challenging. Testing drug sensitivity and selecting more sensitive antitumor drugs for the treatment of gastric cancer patients is of great significance in improving therapeutic benefit. Cells cultured in two dimensions have certain limitations regarding reflecting cancer heterogeneity and the microenvironment. The emergence of the single-cell patient derived organoids (PDOs) transcriptome not only compensates for the deficiencies of traditional cell models, but also provides good results for drug sensitivity tests of gastric cancer, which is a very promising tool for drug sensitivity testing (85, 86).

In conclusion, scRNA-seq may offer an opportunity to find the best treatment combinations based on their intrinsic characteristics of their drug responses. It has the potential to be used for predicting drug sensitivity and screening drugs in the future.

## 4 Discussion

**Figure 2:**
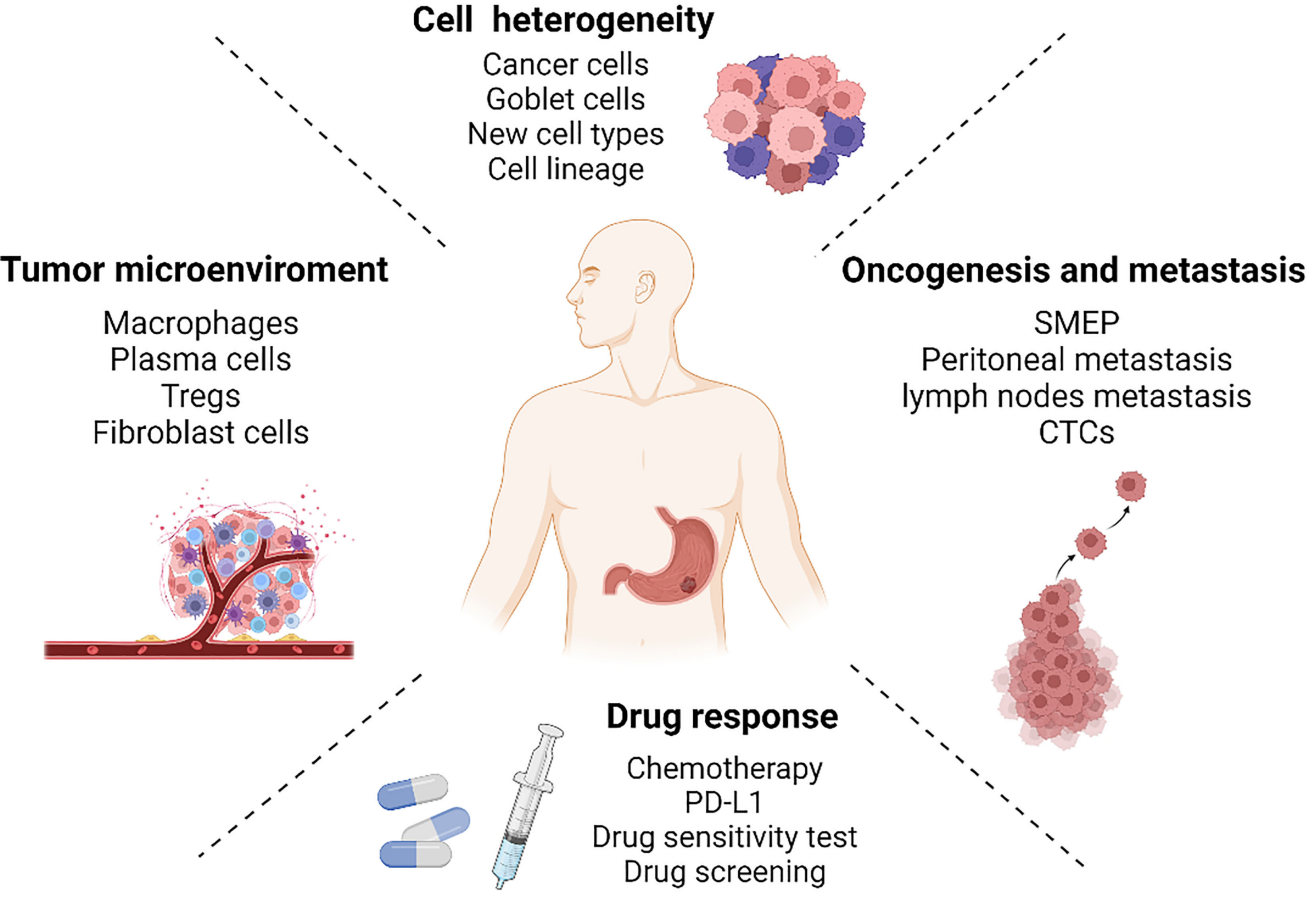
Single-cell RNA sequencing reveals gastric cancer in four aspects: cell heterogeneity, tumor microenvironment, oncogenesis and metastasis, and drug response. Created with Biorender.com.

Gastric cancer is a malignant tumor of the digestive system whose heterogeneity has a significant impact on the clinical outcome and prognosis of patients. With single-cell RNA sequencing, we have gained a deeper understanding of the complexity and diversity of gastric cancer. It is capable of revealing cell heterogeneity, the tumor microenvironment, oncogenesis and metastasis, as well as drug response in an unparalleled manner (**Figure 2**). Even though scRNA-seq is powerful, it can be improved. For instance, not all samples can be processed in an optimal timeframe after being collected. The sequencing results obtained from some frozen samples revealed that the poor cell activity invariably affected the subsequent analysis. Fortunately, cell fixation and preservation methods can now be used in library construction for frozen tissues (87, 88). By using single-nucleus transcriptome sequencing (snRNA-seq), libraries can be constructed on frozen tissues and sequencing results are more consistent than scRNA-seq (89, 90). Some analytical techniques still have limitations. The method of estimating copy number variation based on genetic variation, for instance, is not entirely accurate. Single-cell DNA sequencing retains its irreplaceable advantages in determining copy number variations (48). Furthermore, the emergence of spatial transcriptomes can compensate for the loss of spatial information caused by dissociation of single cell (91).

In future studies, it is anticipated that different types of gastric cancer will be investigated using scRNA-seq in terms of their molecular characteristics, carcinogenesis mechanism, immune response, and drug response. As a result, biomarkers and therapeutic targets for various types of gastric cancer will be discovered, which will facilitate more precise treatment. New biomarkers are expected to become indicators for monitoring, and the discovery of new targets offers more options for precisely treating gastric cancer. Moreover, instead of conventional sequencing analysis, scRNA-seq will probe deeper into the molecular mechanism of gastric cancer. Further molecular and cellular verification is required for results of scRNA-seq in gastric cancer. As an example, the key ligand receptor pairs uncovered through cell communication analysis, how they work, and whether they can be used to treat cancer. New perspective on the molecular mechanisms of gastric cancer can be gained through scRNA-seq. With the improvement of scRNA-seq and the universal of analysis technology, we will see scRNA-seq sequencing used more as part of research evidence, in determining molecular characteristics and lineage relationship of cell populations (92), and verifying gene expression in specific types of cells (93, 94). Additionally, multiomics combined analysis at the level of single cell will reveal the occurrence and evolution of gastric cancer through transcriptomics, genomics, proteomics, and epigenetics. It will be more feasible to support this with bioinformatics analysis techniques (95).

Finally, although scRNA-seq will take some time to become widely used in clinics for a variety of objective reasons, it is extremely promising. In addition to providing more opportunities for gastric cancer research, scRNA-seq will help solve many clinical challenges, advancing the processions of personalized medicine.

## Author contributions

GD substantially contributed to the concept and outline of the work. GD, YC, XZ, SCL, and SL drafted revised the review. ZY and ML revised the review. All authors contributed to the article and approved the submitted version.

## Glossary

**Table:**

scRNA-seq	single-cell RNA sequencing
RNA-seq	RNA sequencing
TCGA	The Cancer Genome Atlas
ACRG	Asian Cancer Research Group
FACS	fluorescence activated cell sorting
snRNA-seq	single-nucleus RNA sequencing
UMI	unique molecular identifiers
CNV	copy number variation
Tregs	regulatory T cells
EGC	early gastric cancer
EBV	Epstein-Barr virus
IGC	intestinal gastric cancer
DGC	diffuse gastric cancer
HDGC	hereditary diffuse gastric cancer
TLSs	tertiary lymphatic structures
CAFs	tumor-associated fibroblasts
SPEM	spasmolytic polypeptide-expressing metaplasia
PC	peritoneal carcinomatosis
EMT	epithelial mesenchymal transition
PD-1	programmed cell death receptor 1
NAG	non-atrophic gastritis
CAG	chronic atrophic gastritis
CG	chronic gastritis
IM	intestinal metaplasia
GC	gastric cancer
AGC	advanced gastric cancer
PBMC	peripheral blood mononuclear cells
CTCs	circulating tumor cells
PDO	patient-derived organoids
TAMs	tumor associated macrophages
NA	not available.
